# Craniofacial superimposition: a review of focus distance estimation methods and an extension to profile view photographs

**DOI:** 10.1007/s00414-022-02871-5

**Published:** 2022-08-24

**Authors:** Carl N. Stephan, Sean Healy, Hamish Bultitude, Chris Glen

**Affiliations:** 1 Laboratory for Human Craniofacial and Skeletal Identification (HuCS-ID Lab), School of Biomedical Sciences, The University of Queensland, Brisbane, 4072 Australia; 2 School of Biomedical Sciences, The University of Queensland, Brisbane, 4072 Australia

**Keywords:** Forensic anthropology, Craniofacial superimposition, Photographic superimposition, Video superimposition, Lens, Focal length, Camera, Photography

## Abstract

Craniofacial superimposition concerns the photographic overlay of skulls and faces, for skeletal identification. As a phased method that depends on photographic optics first and anatomical comparisons second, superimposition is strongly underpinned by the physics of light travel through glass lenses. So that the downstream (and dependent) anatomical evaluations are not thwarted or erroneous identification decisions risked, it is critical that the optical prerequisites for valid image comparisons are met. As focus distance sets the perspective, the focus distance used for skull photography *must* be matched to that used at face photography, so that anatomically comparable 1:1 images are obtained. In this paper, we review the pertinent camera optics that set these nonnegotiable fundamentals and review a recently proposed method for focus distance estimation. We go beyond the original method descriptions to explain the mathematical justification for the *PerspectiveX* algorithm and provide an extension to profile images. This enables the first scientifically grounded use of profile view (or partial profile view) photographs in craniofacial superimposition. Proof of concept is provided by multiple worked examples of the focus distance estimation for frontal and profile view images of three of the authors at known focus distances. This innovation (1) removes longstanding trial-and-error components of present-day superimposition methods, (2) provides the first systematic and complete optical basis for image comparison in craniofacial superimposition, and (3) will enable anatomical comparison standards to be established from a valid grassroots basis where complexities of camera vantage point are removed as interfering factors.

## Introduction

Craniofacial superimposition has long been used in forensic science to assist identification of human skeletal remains when mainstream methods of DNA, radiographic comparison, and fingerprints cannot be used [1–14]. To be employed, craniofacial superimposition requires an intact skull and a facial photograph (taken in life) of the person to whom the comparison is desired and/or to whom the skull is suspected to belong. The skull is photographed at the same orientation as the face, either using still-frame or motion picture photography, and the two images are registered at partial image transparency (superimposition) to assess their degree of anatomical correspondence [1–15].

There are two prime starting premises for craniofacial superimposition: (1) that the anatomy of the face is sufficiently different between individuals that one individual can be differentiated from another (faces are specific to individuals) and (2) skulls and faces belonging to different persons cannot be confused as being derived from the same person (skulls are specific to faces of individuals). Both of these items largely hold true when genetically unrelated individuals are considered, but there is a segment of the population where differentiation based on facial morphology alone is known to be much more difficult (i.e., for genetically identical twins, same sex siblings, and occasional doppelgängers) [16–20]. Here, it is important to note that exceptions to the starting premises may apply as much to skull morphology as they do to facial characteristics, but data concerning the distinctiveness of skulls—as the specific subject matter—are much more sparse than for faces [16–20]. In these discussions of human anatomy patterns, it should be noted that the ability to biometrically differentiate between faces or skulls also depends specifically on what variables are considered as the unit of comparison [18, 20–25]. This factor yields a range of identification potencies for superimposition depending on which particular comparison criteria are chosen.

As for other morphology-based identification methods, a single morphological discordance at analysis is, in principle, enough to discount a skull as a match to a face in craniofacial superimposition no matter what other concordances might exist [26]. For a match call, the skull and face must match in their entirety, whereas for an exclusion call, only a single discordance is required. This establishes an asymmetry in the identification task, per the criteria number for each result, that theoretically makes it easier to discover non-matches than matches [26]. It should also be noted that when multiple different individuals comprise a sequential lineup for a superimposition comparison to a skull, most individuals will by default be ground truth exclusions since only one person in the lineup can ever correspond to a ground truth match. All else being equal, this again makes it easier (by chance alone) to discover non-matches than matches because the former are much more frequent. These factors are important to appreciate in the context that the exclusionary power of the superimposition method is often claimed to be a key attribute [9].

Irrespective of what exact variety of craniofacial superimposition method is employed, there are some generic and widely accepted preferences with regard to the photographs used. These should be in-focus high-resolution images and, in regard to antemortem reference face images, should (1) be taken in close proximity to the time of death, (2) comprise multiple photographs of the same person in different face views, and (3) ideally display anterior dentition with unobstructed line of sight [15]. It is worth noting here that the anterior dentition provides a unique and direct window to the skull in a living person, and, for this reason, visibility of anterior dentition in the facial photograph and physical inclusion of teeth with the skull are highly valued [27–29].

One of the major advantages of the craniofacial superimposition method is its use of facial photographs as the comparative antemortem reference data—these facial photographs are commonly available and often more easily obtained than DNA or medical records [30, 31]. Nevertheless, as for all methods used in the identification context, it is critical that craniofacial superimposition methods are valid and deliver correct results, on a reliable basis. Pursuits to improve and refine methods are important because there are multiple and increasingly frequent examples where present-day craniofacial superimposition methods fail in their objective. For example, successful identification results are sporadic in scientific tests [14, 32–35], and erroneous casework findings have been elucidated through other independent identification lines, such as genetic tests [36–39] and/or radiographic comparisons [37, 38]—this is highly problematic. While methods have commonly been regarded as controversial on the basis of varied results of laboratory tests and personal views [40, 41], wrongful identification results in real-life casework [36–39] confirm without any ambiguity that current methods are plagued by underlying methodological flaws that require proactive redress.

**Fig. 1:**
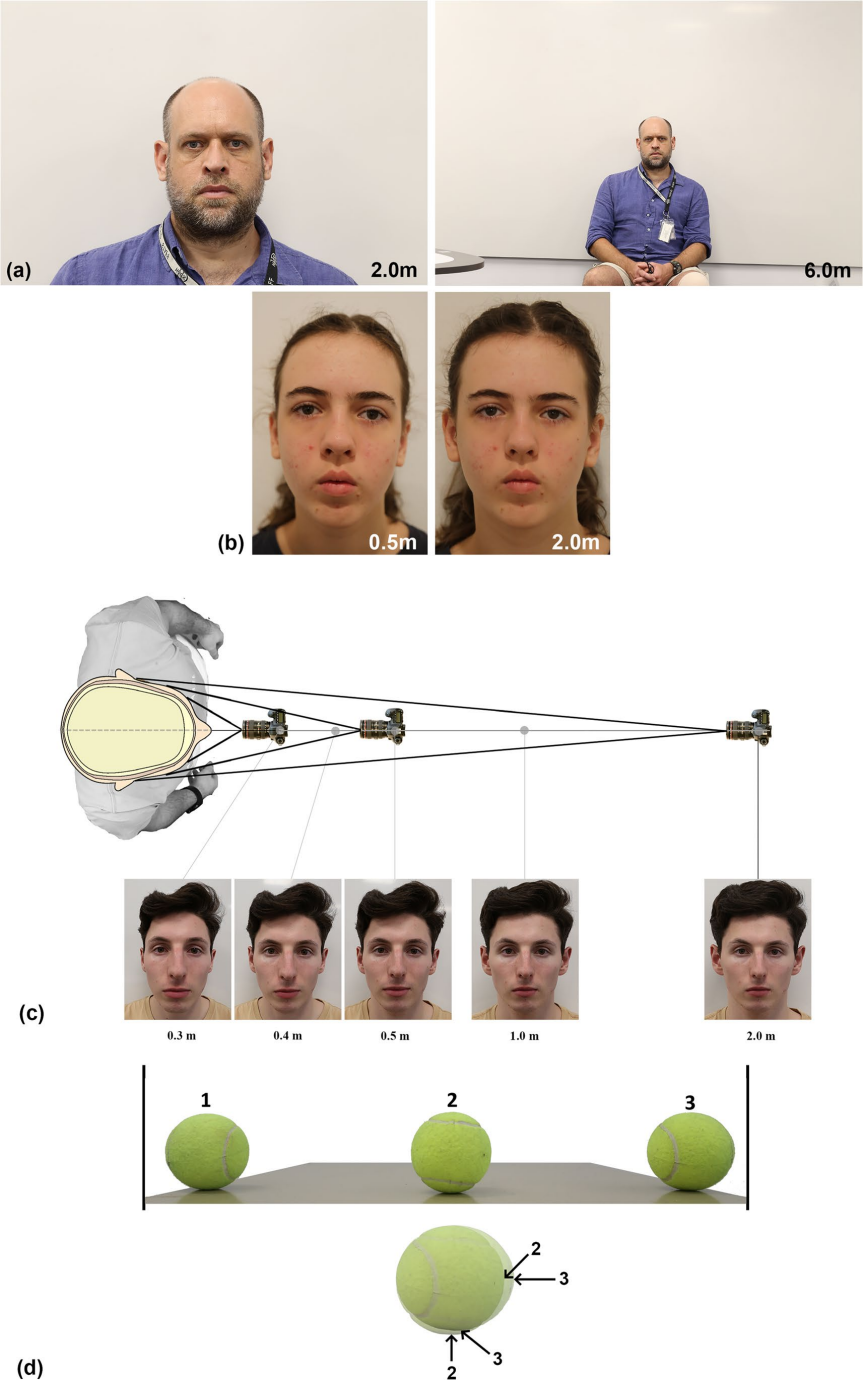
Differential perspective of the same face due to differences in camera vantage point. **a** Perspective impacting subject scale. These two images of the same subject are taken moments apart using the same Canon® EOS 6D camera with the same Canon® macro EF 100-mm prime lens, but at different focus distances (2 and 6 m, respectively). Note that the second image includes more background in the field-of-view, which also makes the head size smaller. **b** Perspective effecting magnification of different parts of the same object, based on their distance to the camera. Note here that at shorter focus distances (such as the left image taken at 0.5 m), the sizes of the mid-face features like the eyes and nose are enlarged compared to the rest of the face and morphologies acquired using longer focus distances (such as the 2.0-m example provided adjacent). The second image at 2.0-m has been taken immediately after the 0.5-m image with almost no delay and enlarged (fixed aspect ratio) so that the trichion to menton distance approximates that for the 0.5-m photograph. Both images are taken using the same Canon® EOS 6D with a Canon® EF 24–105-mm f/4L IS II USM zoom lens. **c** Perspective determination of what counts as the edge of an object. All images taken with a Canon® EOS 6D using the same Canon® EF 24–105-mm f/4L IS II USM zoom lens, but at different focus distances. **d** Perspective giving rise to object stretch at the edges of the photograph due to point projection of the 3D scene using three strategically placed tennis balls, one at the center and one at the left and right edges of the field-of-view (top panel). Superimposition of balls 2 and 3 is shown in the bottom panel, aligned on the right ball margin and with arrow indicating respective ball edges. Note that tennis ball 3 is both wider and shorter in height than ball 2, even though the balls are of the same size in real life. Image taken using Canon® EOS 6D with a Canon® EF 24–105-mm f/4L IS II USM zoom lens

The reliance of the craniofacial superimposition on 2D facial photographs makes the basic physics of light travel through glass lenses critical. While it is well-appreciated in the craniofacial domain that the orientation of the skull must replicate the face for successful superimpositions, other vantage point factors specific to the camera have been paid far less attention, despite being equally fundamental. For example, the subject-to-camera distance or focus distance is set by the camera vantage point, and it plays a critical role in determining the perspective. Perspective is pivotal because it influences how the structure of the skull/face is recorded, i.e., its anatomy on the photograph. When the face is centered within the field-of-view of the camera, perspective has three effects: (1) it determines the absolute scale or size of the skull or face in the camera field-of-view; (2) it determines the relative size of facial features in comparisons to one another; and (3) it determines what parts of a 3D curved surface register as a feature “edge” on a 2D image [7, 31, 42–44] (Fig. 1). If the face is not centered in the field-of-view, then morphology stretching along the film plane and especially at the field-of-view edges (due to point projection) becomes a fourth additional factor to consider (Fig. 1d or for more in-depth explanation [43, 45]), and it will interact in complex ways with each of the aforementioned three items. These modifications to the facial representation carry major ramifications for the downstream morphological comparison (that is anatomy based) and subsequently any identity determination that the superimposition will ultimately yield.

**Fig. 2:**
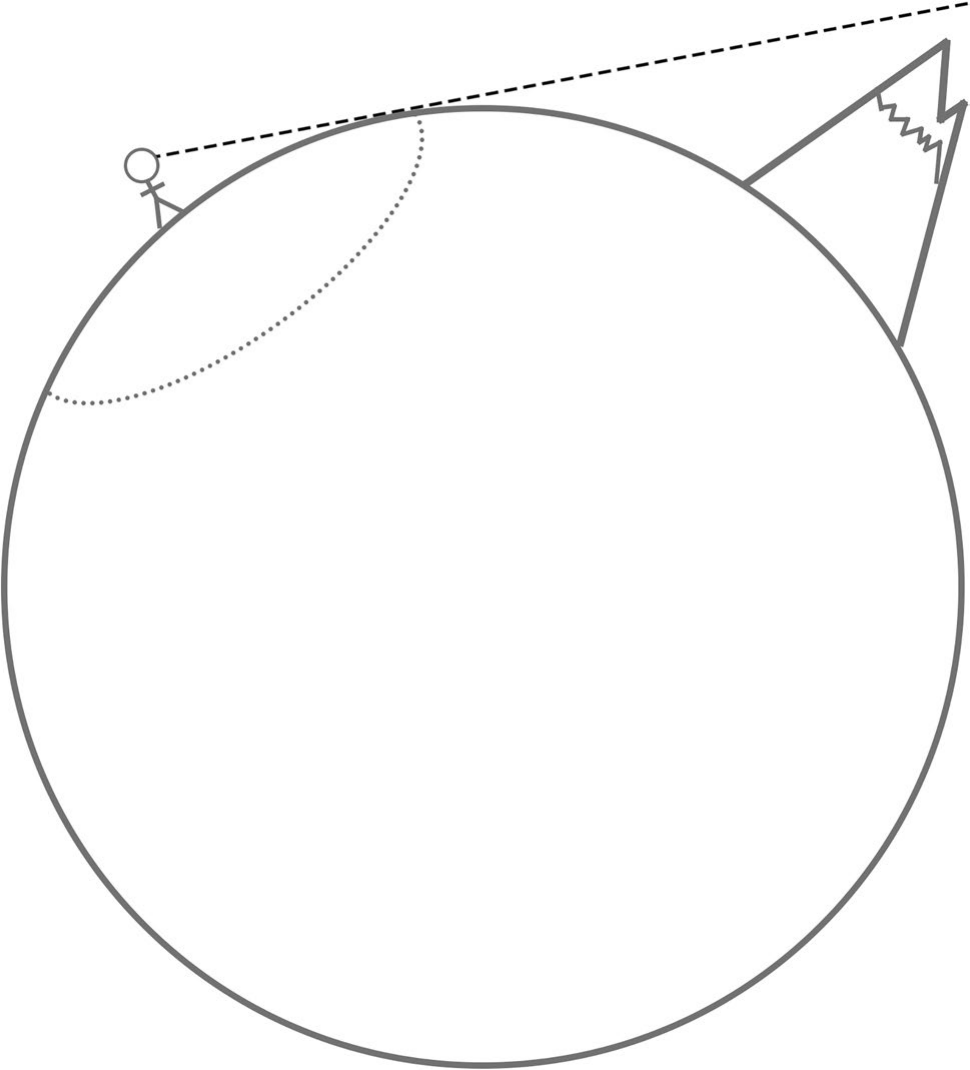
A simple example of a horizon (dotted line) on a curved spherical surface. In this case, the subject cannot see the mountains for the plains! The mountains fall beyond the line of sight (dashed line). If the subject changes position, so does their horizon and view of the edge of the earth. The same applies to vantage points with respect to face and, for example, visibility of the ears and sides of the face in Fig. 1

While all four abovementioned factors are critical to the projected recording of the face and skull on the image sensor, it is worth highlighting that factor 3 (what parts of a 3D curved surface register as a feature “edge” on a 2D image) is analogous in many respects to horizons. On curved spherical structures, such as the earth, what you can see depends on your vantage point or line of sight, and it subsequently sets the local horizon (Fig. 2). You cannot see features that fall behind the horizon (even though they are there), and horizons will change as the vantage point changes. That is, as one travels to a different viewing location or vantage point, the landscape structure changes. The same applies to the 3D spherical structure of the skull and face in portrait photography (Fig. 1).

There have been some isolated calls since the 1970s, for superimposition practitioners to consider the importance of perspective and perspective distortion [42, 46], but generally, these calls have waned as focus distance has been found to be too difficult to estimate from a facial photograph alone. In fact, so difficult was it found to be that some practitioners labelled focus distance estimation an “impossible” task ([12] p. 122–3, [47] p.118, [48] p.240). Continued use of methods in casework without any science-based ability to estimate the focus distance is, subsequently, a major concern. Consistent findings of the method’s unreliability in scientific tests [14, 32–35] and erroneous casework findings verified by independent identification lines (such as genetic tests [36–39] or radiographic comparisons [37, 38]) are testament to this problem. These cases of mistaken identity implore the derivation of focus distance estimation methods to eliminate error arising from the use of arbitrary, guessed, or inapplicable subject-to-camera distances [43].

Recently, it has been elucidated that the focus distance is, in fact, not impossible to estimate from fontal view face photographs; rather, it can be done and with measurable estimation errors [31, 44]. This represents a breakthrough; however, the extension of these estimation methods to profile or partial profile image views has not been forthcoming. It is critical that this hurdle be overcome since in the casework context, multiple face views enable several superimpositions to be undertaken for the same subject, thereby improving reliability and confidence in the superimposition result [49].

In this paper, we review the pertinent camera optics applicable to focus distance estimation in craniofacial superimposition for the first time in the literature. We provide a step-by-step explanation of how and why the new focus distance estimation method of *PerspectiveX* works and describe a novel extension to faces rotated relative to the line of sight of the camera. We provide multiple worked examples of focus distance estimation from such “profile” photographs (of the authors) to demonstrate proof of concept, practical ease, and first insights towards the accuracy of the newly formulated approach.

## Basic camera optics pivotal to craniofacial superimposition

**Fig. 3:**
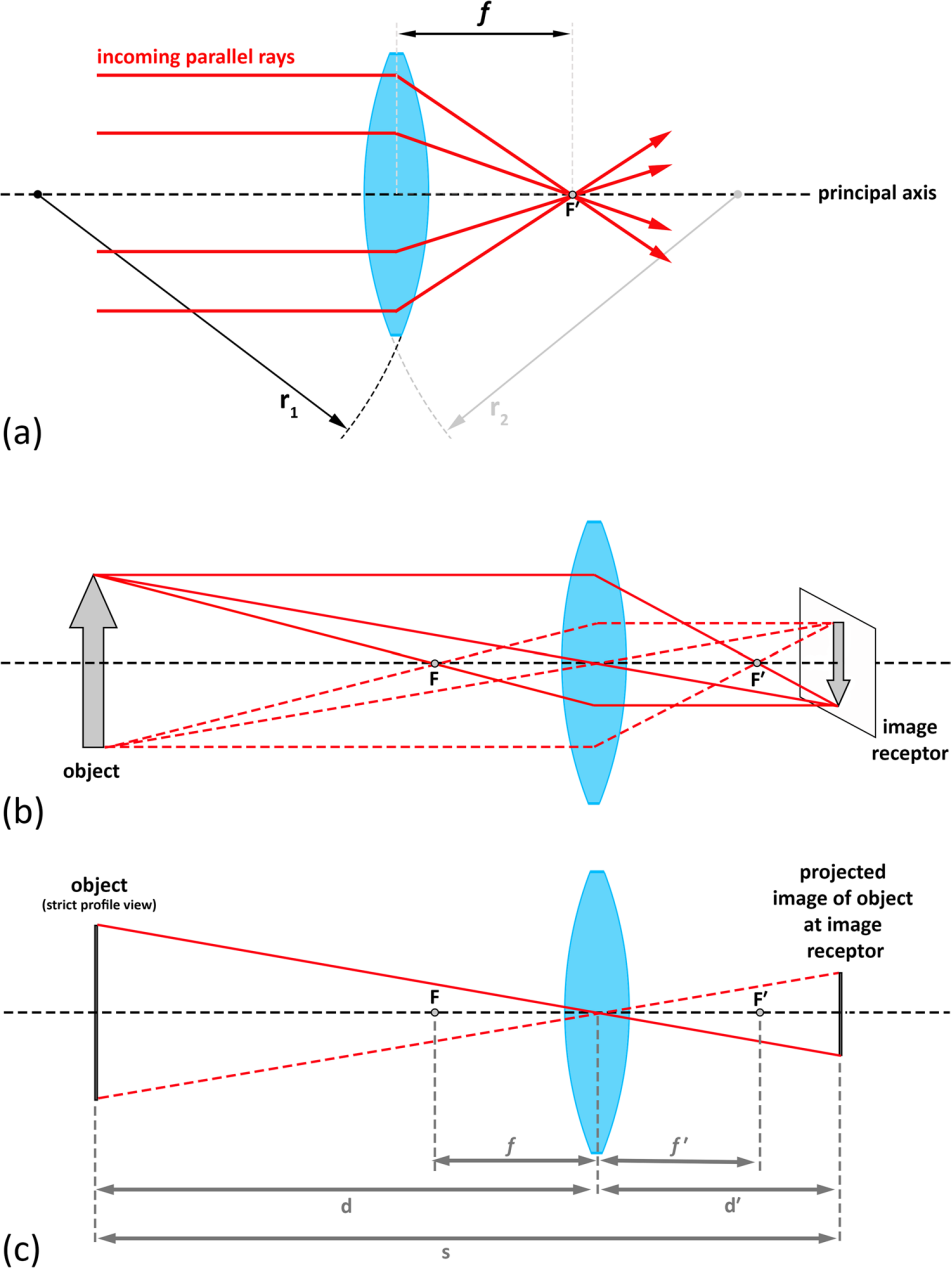
Thin lens optics relevant to craniofacial superimposition. **a** Focusing of light by a thin lens with negligible thickness compared to the radius of the lens’ curved surfaces (*r*_1_ and *r*_2_). *f* = focal length. *F*′ = focal point. **b** Point projection of an object (gray arrow), via a thin lens, to an image receptor plane. *F* = the front nodal point. *F*′ = the rear nodal point. Example light rays are traced for two extreme ends of the arrow. Three rays are traced from one end of the object as solid red lines, while rays from the opposite end are traced as dotted lines. Note that there are two focal points where the light rays converge. One is in front of the lens (front nodal point), and the other is to the rear of it (rear nodal point). **c** A simplified representation of **b** showing the object and image receptor from strict profile views, with fewer rays, and with pertinent distances illustrated: *d*, the working distance; *d*′, image distance; and *s*, the focus distance. The focal length is represented by both *f* and *f′* (*f* = *f′*)

With regard to camera systems, it should be noted that precisely tracing light rays through camera lenses is a complex undertaking, in part because modern-day cameras use multiple lens elements to tweak rays and correct for aberrations that exist within single lenses and camera systems. Subsequently, camera optics are commonly reduced to a “thin lens” model, meaning that the optical complexities introduced by the thickness of the lens are ignored to simplify the descriptions and mathematical calculations. A “thin lens” is defined as one that has a thickness, which is negligible compared to the much larger radii of its curved lens surfaces (Fig. 3). This simplified thin lens model is so fundamental that its basic principles are widely acknowledged to be applicable to more complex camera systems that use multiple lenses, making it a common reference standard in photography [50]. It is also the model employed in this paper.

Since light travels faster in air than in glass, the glass lens bends (refracts) the light, such that incoming parallel light rays (emitted from an object) are brought together at a single point. This point is called the focal point (F′), and the distance from the lens to this node of focus, when the lens is focused at infinity, is called the focal length (*f′*) (Fig. 3) [50] (note here that the focal length is not the same as the focus distance). The light rays from an object are not always parallel, and it is important to note that light travels in both directions through the lens (not just front-to-rear but also rear-to-front), thereby producing two focal points, one in front of the lens (F) and one behind (F′) where the light rays converge (Fig. 3) [50]. These focal points are termed the front and rear nodal points, respectively [50].

For a thin lens camera, *with the object size on the image receptor the same as the object’s physical size in real life*, the focal length is the same as the distance from the rear nodal point to the image receptor [50]. However, in modern-day cameras, the image receptor is much closer to the rear nodal point, producing a smaller representation of the object at the image plane. Subsequently, the distance from the lens to the image receptor, commonly known as the image distance (d′), is similar in size to the focal length (*f′*), especially when their difference is considered relative to the large whole meter distances that accompany subject placement in front of the camera. Effectively, this renders *f′* and d′ to be equivalent.

**Fig. 4:**
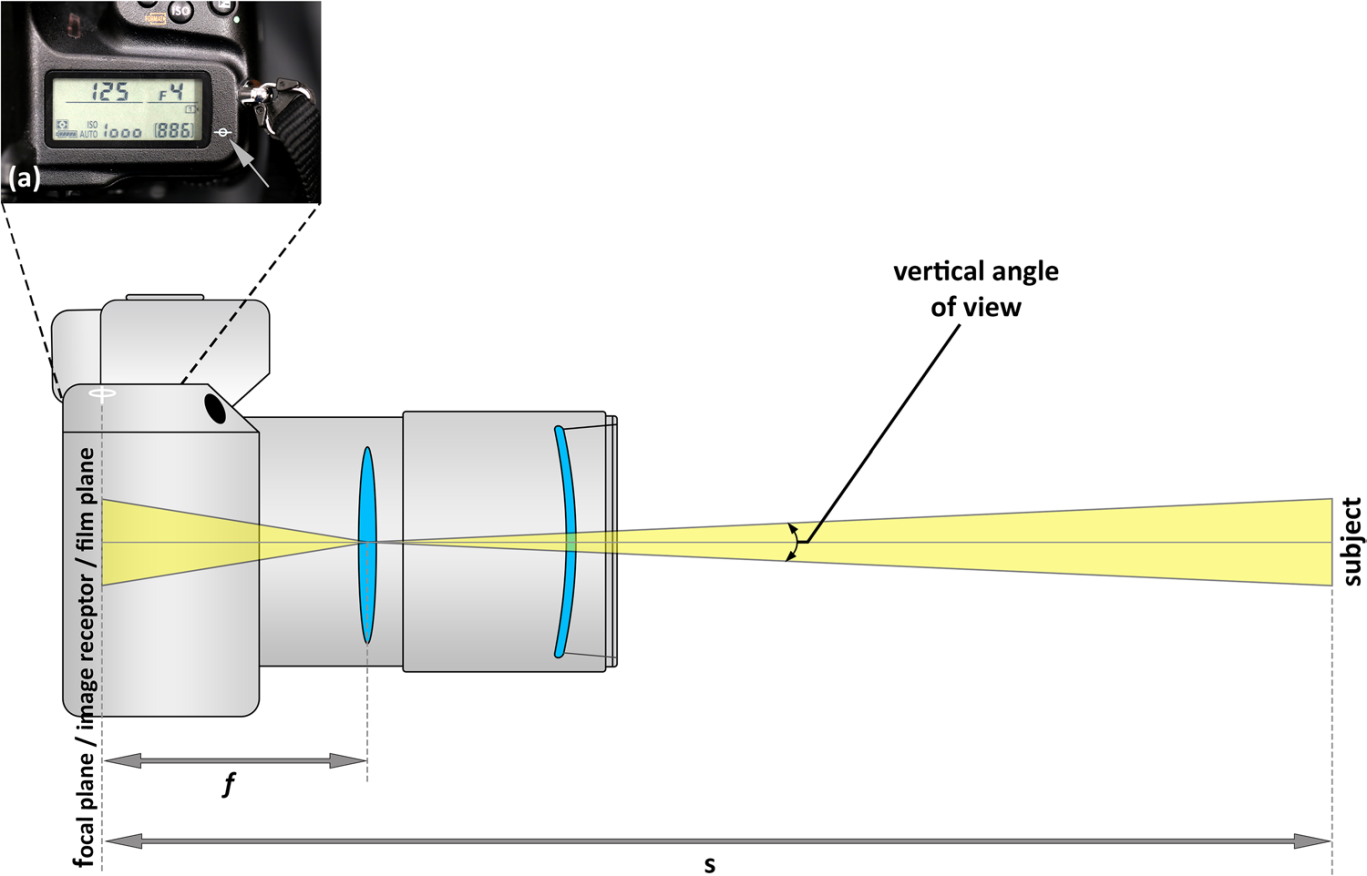
Simplified schematic of DSLR optics, showing the vertical angle of view, the focal length (*f*), the focus distance (*s*), and the image receptor plane marked by the focal plane indicator (*ϕ*). The inset **a** shows the focal plane indicator on a real DSLR camera viewed from above (the front of the camera is towards the top of the image within the inset)

The focal length sets the angle of view, which determines the amount of the 3D scene that is seen and recorded by the image receptor (Fig. 4; Table 1) [50]. A short focal length lens provides a large field-of-view with little magnification, whereas a long focal length provides a narrow field-of-view with large magnification [50], hence the photographic nomenclature of wide-angle and telephoto lenses, respectively.

To be specific, the distance from the subject to the image receptor is termed the focus distance (s) or the subject-to-camera distance (SCD). In the thin lens camera model, the focus distance is equivalent to the image distance (d′) plus the working distance in front of the lens (d) (Fig. 3c), such that:

1 $$\mathrm{s}={\mathrm{d}}^{\mathrm{^{\prime}}}+\mathrm{d}$$

If a small error tolerance is provided, then the focus distance can be approximated as the sum of the focal length (*f′*, which is close to equivalent for d′ as described above) and the working distance in front of the lens (d) (Fig. 3c), such that:

2 $$\mathrm{s}={f}^{^{\prime}}+\mathrm{d}$$

In regard to the camera sensor, a common standard size is the “full-frame” drawn from the 36 × 24 mm standard size used for 35-mm wet-film photographics [50]. Many digital single-lens reflex (DSLR) cameras hold such full-frame sensors. Cameras with sensor sizes smaller than the full-frame size are termed “cropped sensors,” since they record less of the 3D scene. For single-lens reflex (SLR) cameras, which provide for more than just “point-and-shoot” functionality, the position where the 2D imaging sensor exactly crosses the focal axis is marked by an uppercase Greek phi (*ϕ*) on the external camera body housing (Fig. 4). This landmark (called the *focal plane indicator*) serves as a convenient and easy reference to determine the focus distance where needed or applicable. The importance of the focal plane indicator is highlighted in cinematography where this marker often has a tab or notch on the camera that enables a standard tape to be clipped directly to the camera to enable manual focus distance measurement, so that precise focus can be pulled for a scene.

If the very small focal length adjustments that occur with focusing are ignored, prime lenses (also known as fixed focal length lenses) have a single set focal length. This length, determined by the physical properties of the lens and its construction, is termed the *effective focal length*, and it is this variable that represents *f′*. It is noteworthy that lens manufacturers often round the effective focal length up by a small amount to whole integer *nominal focal lengths* for the convenience of sales and marketing [31]. Typically, the nominal focal lengths are within 5% of the effective focal lengths, and they are almost always rounded up, not down [31]. In addition, standard “focal lengths” applicable to the 35-mm photography should not be confused for the “35-mm equivalent focal length” since these two items are not the same, despite sounding similar. The latter are used for lenses paired with cropped image sensors, and they represent an attempt to “recalibrate” the lens performance to the full-frame context (even though the sensor size is a not full-frame). This means the crop factor is effectively ignored with the “35-mm equivalent focal length,” making this metric a marketing gimmick employed by camera manufacturers to make the focal length of the lens sound larger and more appealing to potential customers.

A small focal length lens, with a wide field-of-view and little magnification effect, allows the photographer to move very close to the subject, without the subject being too large for the preset field-of-view. This introduces large perspective changes of the subject, due to the very short focus distance (Fig. 2b/c). Here, it is important to note that the focus distance, not the lens, creates the perspective. Long focal length lenses produce the same perspective at short distances, but because their zoom is larger (such that less of the scene can be visualized), the perspective changes are less noticeable even though they are still present.

## Perspective versus Perspective Distortion

In photographics, the term “perspective” is used to describe the rendering of 3D objects to 2D planes to give an impression of length, width, and depth. “Perspective distortion” is often used to describe the realized 2D projected effect, in contrast to the original 3D object viewed in the 3D real-life environment. Subsequently, the two terms are often used interchangeably as synonyms because they describe all four camera vantage point–induced peculiarities previously mentioned:

- 1. Scale of the subject respective to the rest of the scene (Fig. 1a)
- 2. Size of subject features in relation to one another (Fig. 1b), e.g., nose on a face
- 3. What counts for an object’s peripheral edges or horizons (Fig. 1c)
- 4. Stretching of the subject along the 2D film plane (Fig. 1d) [50]

In craniofacial superimposition, there is utility in differentiating which of the perspective-induced factors can be corrected by simple fixed aspect ratio scaling (i.e., effect 1 above), in contrast to the other effects that cannot be (effects 2–4). Subsequently, for this paper, we define perspective to collectively refer to *all four of the abovementioned effects*, while perspective distortion is expressly reserved *only for the last three factors (2–4)*. These perspective distortion factors (2–4) are especially problematic for craniofacial superimposition because they cannot be compensated by simple fixed aspect ratio image scaling.

## What Causes Perspective Distortion?

**Fig. 5:**
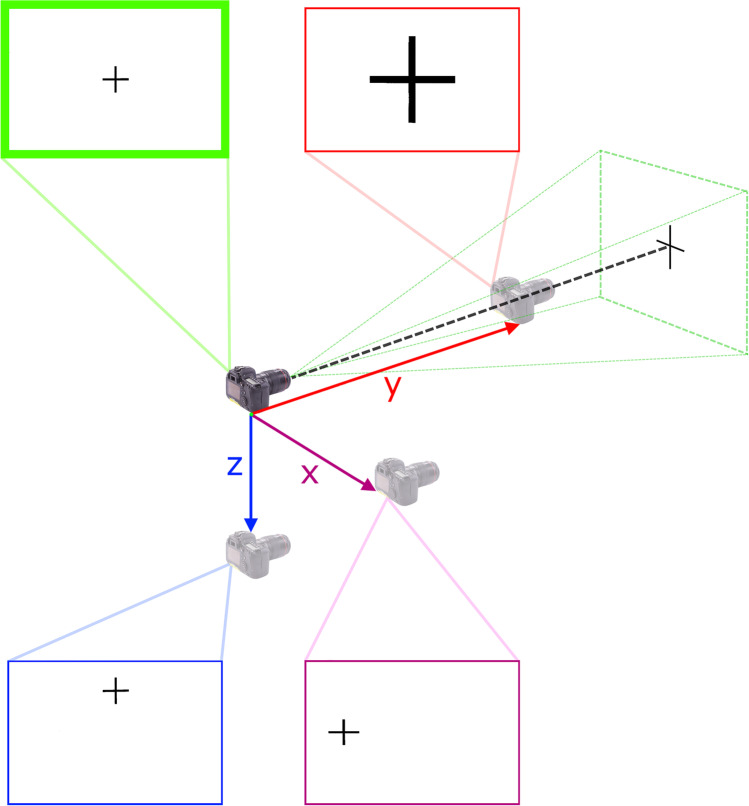
Coordinate positioning of the camera along *x*-, *y*-, and *z*-axes relative to the subject (a 2D cross) and the resultant 2D representation in the output photographic images. Note the enlargement of the cross on the image receptor with travel towards the subject on the *y*-axis and the shift in the position of the cross towards the edges of the field-of-view with travel along the *x*- and *z*-axes (that results in stretching across the film plane—see Fig. 2d)

Perspective distortion is a consequence of the vantage point of the camera (i.e., its *x*, *y*, and *z* positions in space relative to the subject) and the projection or rendering of the 3D scene to a 2D plane [43, 45, 50]. For effects 2 and 3 mentioned above, the subject-to-camera distance (y distance) is the primary single determinant (Fig. 5). That is, as the camera moves closer to the subject, parts that fall closer to the camera (shorter subject-to-camera distance) are magnified in comparison to those that fall further away (Fig. 1b/c). Additionally, parts that are closer to the camera can block line of sight to other parts that are further away (Fig. 1c). This means that it is not only the 3D head structure that defines the on-image head anatomy, *but rather the vantage point also contributes, such that the projected anatomy which is recorded on the 2D photograph is a manifestation of both factors.* For effect 4 (Fig. 1d), z- and x-axis travels are the primary determinants (Fig. 5).

Perspective distortion effects are most evident when a short subject-to-camera distance is employed with a wide-angle of view (Fig. 1). Of course, where very short focus distances are employed with ultrawide-angle lenses, the perspective distortion is even further exaggerated. This applies to smart phones because the shallow depth of the phone demands the use of ultrawide lenses.

## Why Focal Length Is Not Responsible for Perspective Distortion

The focal length of the camera lens determines how much of a 3D scene fits in the field-of-view or, in other words, the zoom. The glass of the lens does not determine the vantage point of the subject, rather the physical position of the camera relative to the subject does (i.e., the focus distance or the SCD). Subsequently, it is not the focal length of the lens but rather the camera position (and only the camera position) that determines the perspective distortion [50].

**Fig. 6:**
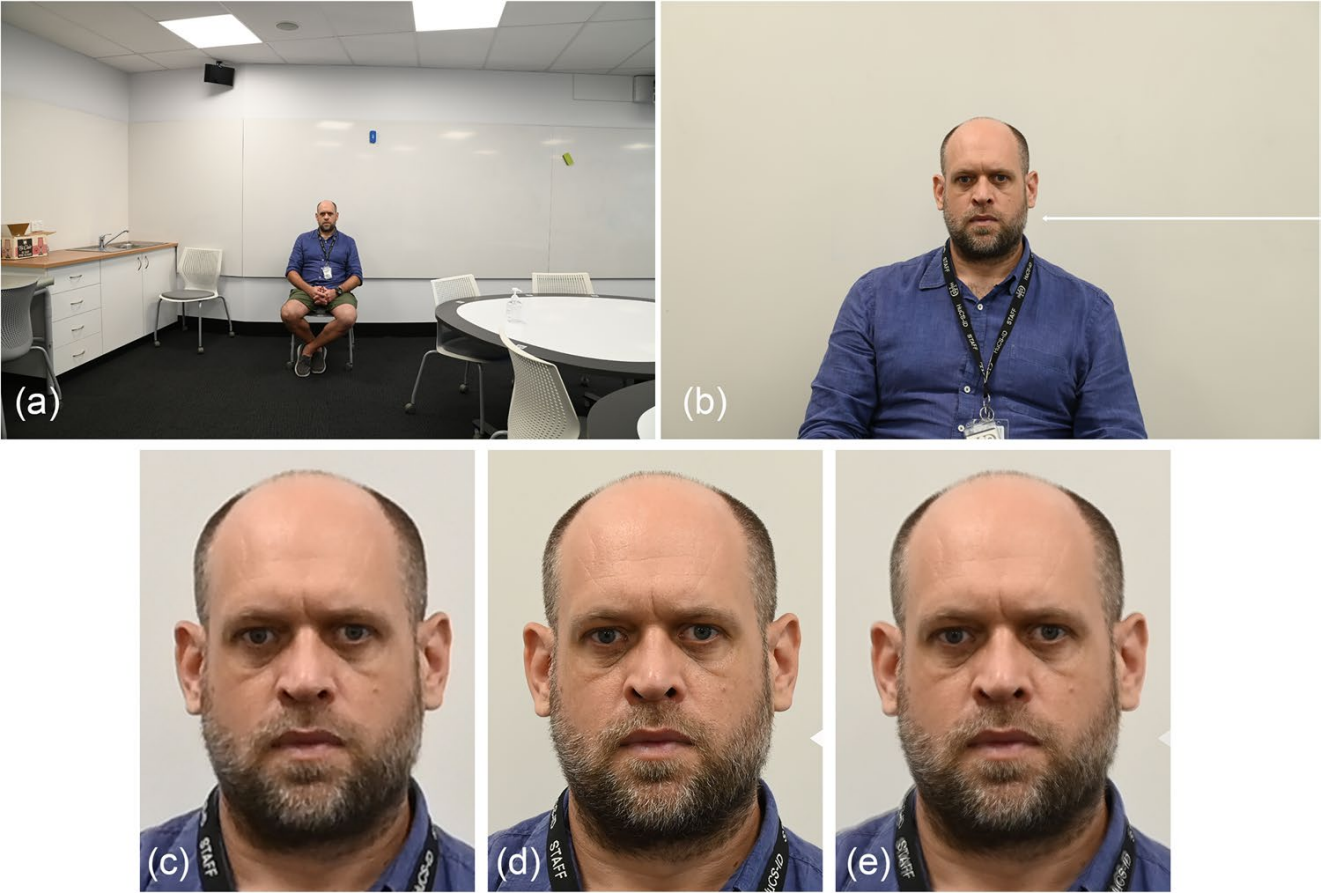
Images of the same subject taken using the same Nikon® D780 24.5-megapixel camera at the same focus distance (3.5 m), but with at two different focal lengths (24 and 120 mm on an AF-S NIKKOR® 24–120-mm 1:4G lens) to show that the perspective distortion is set by the focus distance, not set by the lens/its focal length. As the focus distance has not been changed, after fixed aspect ratio resizing of the image taken with the shorter focal length lens, the two faces precisely superimpose. **a** Raw image produced by the macro 24-mm lens. **b** Raw image produced by the 120-mm lens; note the white arrow included on the image to differentiate it from **a**. **c** Cropped and fixed aspect ratio magnified view of the face from **a** to match the trichion to menton facial height in **b**. **d** Cropped image to view face from **b**; note the arrowhead marking the **b** image. **e** Superimposition of images **c** and **d**. Note image **c** possesses lower resolution than **d** since it is derived from a wide-angle view lens, but that the images precisely superimpose (perspective distortion is identical)

This can easily be demonstrated by photographs taken of the same scene with a short and long focal length lens at a constant subject-to-camera distance (Fig. 6). In this arrangement, the perspective distortion is the same and unaffected by interchanging the lens to a different focal length. The long focal length lens has more zoom, so what fits in the field-of-view is less, but the perspective distortion is the same as that of the shorter focal length lens (Fig. 6). As mentioned earlier, very long focal lengths (that provide large zoom so that only a highly magnified part of the subject can be seen) create the impression that the perspective distortion is small, but this is just an illusion. The perspective effects are still present; it is just that they are harder to appreciate because the field-of-view of the scene is less [43]. This point is important to emphasize since the lens is commonly confused as the source of the perspective distortion in the craniofacial superimposition [7, 12, 40, 47, 48, 51, 52] when in fact it is the focus distance, not the lens, that is responsible.

**Fig. 7:**
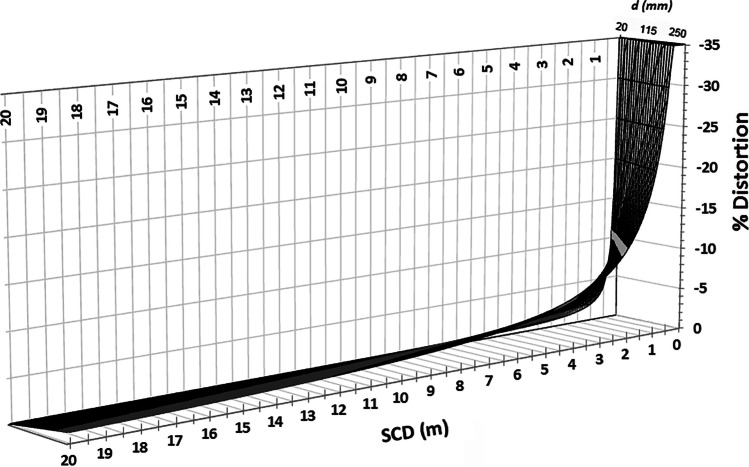
Reverse logarithmic perspective distortion decay curve for two 179-mm lengths, plotted as a function of distance separating the lengths (*d*), after Stephan [43]. Note the cliff-like and very steep increase in the perspective distortion magnitude at short subject-to-camera distances. Figure is reproduced from [43] p. 520.e7 with permission by Elsevier

There is no ideal focal distance or focal length to use for facial photography [50], and which size to use depends on what the photographer desires to record on the photograph and also how they wish to go about acquiring it [50]. In portrait or fashion photography, the photographer may wish to be close to the subject (small subject-to-camera distance) to be able to easily communicate verbal instructions to the subject/model—in these instances, the photographer will select a small focal length lens that has a wide angle of view. At such short subject-to-camera distances, a larger magnitude of perspective distortion will be embedded in the image (due to the short focus distance). It is important to note here that the perspective distortion relationship is not linear. Rather, it follows a reverse logarithmic function meaning that the perspective distortion enlarges rapidly and disproportionately at short subject-to-camera distances [43] (Fig. 7). This makes perspective distortion an especially relevant factor for craniofacial superimposition since facial images with good resolution are often those photographed with smaller focus distances (such as < 3 m).

For two images taken with different focal length lenses at the same camera vantage point (*x*, *y*, and *z* positions), the perspective is not the same, but the *perspective distortion is the same* (see Fig. 6). Fixed aspect ratio enlargement of the subject in the image taken with the smaller focal length lens demonstrates this, by exact alignment with the subject recorded in the image taken with the longer focal length lens (Fig. 6). If different subject-to-camera distances are employed, this relationship does not apply. Adjusting the magnification of one of the images to approximate the other does nothing to resolve the mismatch since the two images hold fundamentally different perspective distortions (see, e.g., Fig. 1b/c). Note here the image taken with the longer focal length provides a higher resolution of the face because the face fills more of the image receptor.

## Why Perspective Distortion is Not Corrected by Zoom Adjustment

Another common misperception in the superimposition literature, especially with video superimposition that utilizes variable zoom lenses, is that the zoom can be tweaked on the camera viewing the skull, so that the skull size is adjusted to approximate the face to “correct” the size mismatch resulting from perspective differences [40, 47, 51, 52]. This is problematic since the operation fails to correct for the last three of the four total perspective relevant factors (i.e., it fails to correct any of the perspective distortion). Perspective and its associated perspective distortion can only be replicated in a second image by employing the same camera vantage point (i.e., focus distance) as used to acquire the first image.

Replicating the camera vantage point controls for all aspects of perspective and perspective distortion factors in one maneuver. The focal length of the lens does not need to be the same for all of the perspective distortion factors to be controlled; however, it is better if the same nominal focal length lens is used, because all four perspective items can then be addressed at the same time (rather than just the three perspective distortion variables).

The nominal focal length of the lens is easily determined from the EXIF data that routinely accompany electronic images and that can be accessed via free online EXIF readers [31, 44]. Once the nominal focal length is determined, it not only sets which lens should be used to photograph the skull, but it can also be used to estimate the effective focal length and, in turn, the focus distance for a full and proper accounting of the image perspective.

## How to Estimate the Focus Distance

To estimate the focus distance, the thin lens model of a camera can be employed along with the rules of similar triangles. According to these rules, it is readily observed (and widely known [46, 50]) that the following ratios are equivalent:

3 $$\frac{object\; height\; on\; sensor ({A}^{^{\prime}})}{focal\; length\; (f)}=\frac{real\; life\; object\; height\; (A)}{distance\; to\; the\; subject\; from\; the\; front\; focal\; point\; (d-f)}$$

Since the distance *f* is generally very small compared to *d*, it can be ignored at the denominator, e.g., per Titlbach’s recommendation [46], to simplify the ratios:

4 $$\frac{object\; height\; on\; sensor\; ({A}^{^{\prime}})}{focal\; length\; (f)}=\frac{real\; life\; object\; height\; (A)}{working\; distance\; in\; front\; of\; the\; lens\; (d)}$$

Subsequently this equation can be reshuffled to solve for the working distance:

5 $$d={f}^{*}A/{A}^{^{\prime}}$$

The object height on the sensor in mm (*A*′) can be calculated by the measurement of the object on the sensor in pixels (*x*) multiplied by the pixel size (*y*), such that:

6 $$d={f}^{*}\left(A/\left({x}^{*}y\right)\right)$$

Since the position of the lens within the camera is not typically marked on the lens housing and too difficult to determine for more complex lenses constructed from multiple lens elements, the focal length (*f*) can be added to the working distance (*d*) to estimate the focus distance:

7 $$\mathrm{Focus\; distance}\left(s\right)=f+{f}^{*}\left(A/\left({x}^{*}y\right)\right)$$

As mentioned earlier, the focus distance is convenient since it corresponds to the optical projection of the scene and because the image receptor position is clearly marked on DSLR cameras making its measurement easy for validation. Equation (6) can be simplified with a multiplier of *f*, yielding the final *PerspectiveX* algorithm [44]:

8 $$SCD=f\left(1+A/\left({x}^{*}y\right)\right)$$

As the real-life size of an object (*A*) is required by *PerspectiveX* for focus distance estimation, a sample mean of the palpebral fissure length (endocanthion [*en*′] to exocanthion [*ex*′]) can be used as a substitute for the individual’s real palpebral fissure length [44]. This is possible because (1) the palpebral fissure length possesses a very small variation range between subjects [53] due to tight evolutionary constraints on the visual system [31, 44] and (2) the palpebral fissure is very close to parallel with the image receptor plane, when the face is centered within the field-of-view for a frontal view image [44].

## A Novel Extension of *PerspectiveX* to Profile Images

To extend *PerspectiveX* to profile (or partial profile) images, all that is needed is a second anatomical character, additional to the palpebral fissure, that can be seen in both the frontal and the profile images. This bypasses the requirement to find another sufficiently invariant anatomical trait to use for profile images alone, which has not been forthcoming [31]. In essence, the palpebral fissure is used to “calibrate” the real-life size of the other facial character that is clearly visible on both photographs (e.g., pupil [*p*′] to stomion [*sto*′] distance measured down the median plane). Once this second dimension is determined, it can serve as the input variable to the *PerspectiveX* formula for the profile view. There is no a priori requirement for the frontal and the profile image to possess the same focus distance; rather, the images can be taken with different focal length lenses and at different focus distances and still be used successfully for the *PerspectiveX* result.

**Fig. 8:**
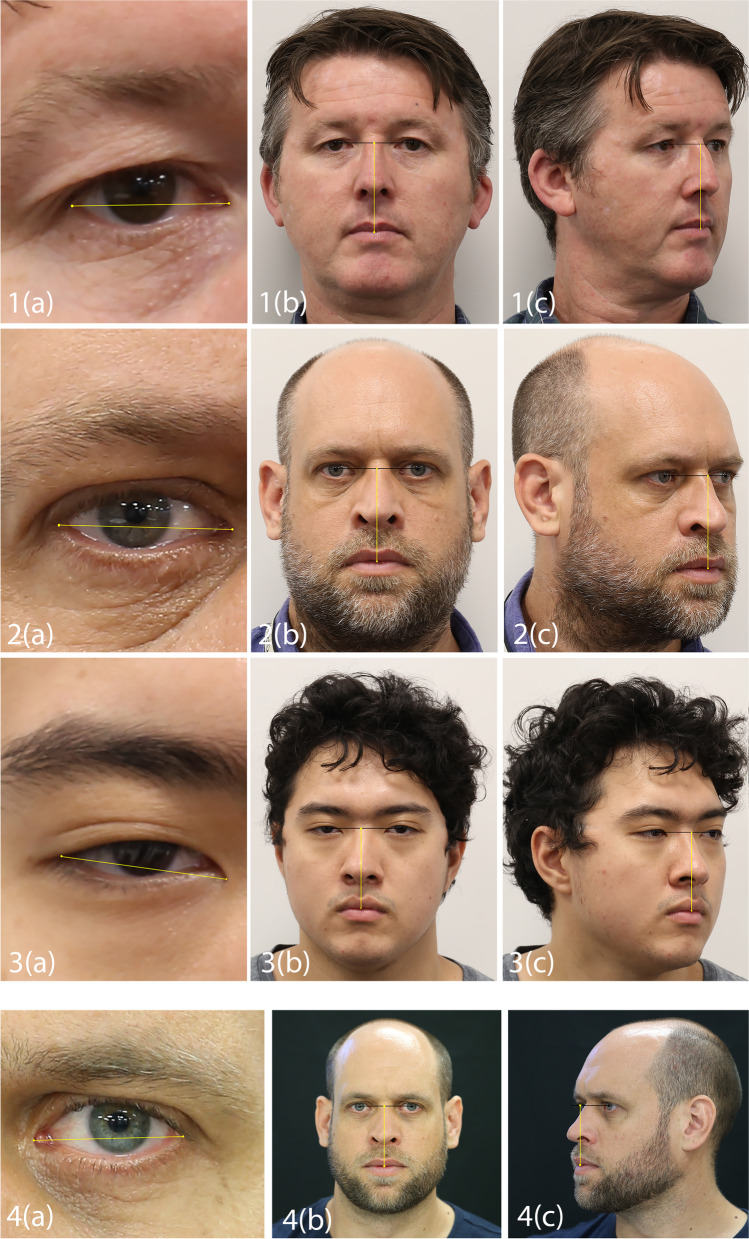
Estimating the focus distance using *PerspectiveX* from frontal and profile view photographs. Panels 1–3 represent example photographs taken at 2-m focus distance of three authors (CG, CS, SH; right side palpebral fissure measured). Panel 4 represents example photographs taken at 2-m focus distance, as previously used in Stephan [44] (left side palpebral fissure measured). All images in this figure have been acquired using a Canon® 6D full-frame 20.2-megapixel camera body fitted with a Canon® IS 2.8-speed 100-mm fixed macro lens. To estimate the focus distance, the following three measurements (highlighted by yellow image bars) are required: **a** palpebral fissure length from a frontal view photograph of the subject (column **a** images); **b** secondary character, such as pupil (*p*′) to stomion (*sto*′) distance in the median plane, from the same frontal view photograph as used for palpebral fissure length measurement (column **b** images); and **c** the same secondary character’s distance (e.g., *p*′-*sto*′) from the profile view photograph (column **c** images)

The step-by-step process is as follows:

1. Measure the on-image palpebral fissure size on the face in the frontal view image.
2. Use the on-image palpebral fissure size (from the frontal view image) and the real-life mean palpebral fissure length value from a sample mean to calculate the real-life size of another facial dimension that is clearly visible in both the frontal and the profile images (e.g., *p*′-*sto*′) and which falls near the plane of the eyes (so it is in or close to the relative parallel zone; see [31]) (Fig. 8). To do this, (a) calculate the ratio of the on-sensor size of the palpebral fissure (mm) to the sample mean (mm), and (b) divide the on-sensor dimension (mm) of the other facial characteristic (e.g., *p*′-*sto*′) by this ratio. The following formulae achieve this:

9 $${PF}_{ratio}= \frac{\left({PFL}_{sensor} * pixel \;size\right)}{{\overline{x} }_{palpebral\; fissure}}$$

10 $${RL}_{{p}^{\mathrm{^{\prime}}}-st{o}^{\mathrm{^{\prime}}}}=\frac{({{p}^{\mathrm{^{\prime}}}-st{o}^{\mathrm{^{\prime}}}}_{sensor} * pixel\;size)}{{PF}_{ratio}}$$

where:

*PF*_ratio_: ratio of the on-sensor size of the palpebral fissure (mm) to the sample mean (mm)

*RL*_p__′-__sto__'_: real-life estimate of the *p*′-*sto*′ distance (mm)

*PFL*_sensor_: on-sensor length of the palpebral fissure (pixel units)

*pixel size*: size of the pixels on the image receptor (mm)

*x̅*_palpebral__fissure_: a sample mean for the adult palpebral fissure such as that provided by Farkas [53] (in mm)

*p*'-*sto*'_sensor_: on-sensor length of the *p*'-*sto*' dimension (pixel units)

3. Substitute the real-life dimension of the secondary character (*RL*_p′-sto'_) into the *PerspectiveX* equation, along with the sensor size of the same dimension (from the profile image), to calculate the SCD for any profile, partial profile, or ¾ view photograph. This method assumes that the head is close to centered in the field-of-view (of both images) and the plane of the face is approximately orthogonal to the line of sight of the camera for the frontal view image. When these conditions are met, the focus distance estimation will be appropriate.

Below is a worked example of the calculation corresponding to the profile image presented in Fig. 8 (3c) that holds a ground truth focus distance of 2.0 m.

***Input measurements/statistics to Eq. ******9******:***

$${PF}_{ratio}= \frac{\left(256.14 * 0.00655\right)}{31.3}= 0.0536$$

where:

*PFL*_sensor_: 256.14 pixels (yellow line measurement in Fig. 8-3a).
*pixel size*: 0.00655 mm.
*x̅*_palpebral__fissure_: 31.3 mm.

***Input measurements/statistics to Eq. ******10******:***

$${RL}_{{p}^{^{\prime}}-st{o}^{^{\prime}}}=\frac{(624.25 * 0.00655)}{0.0536} =76.28$$

where:

*p*'-*sto*'_sensor_: 624.25pixels (yellow line measurement in Fig. 8-3b).
*pixel size*: 0.00655 mm.
*PF*_ratio_: 0.0536.

***Input measurements/statistics to PerspectiveX (Eq. ******8******):***

$$\mathrm{SCD }\left(\mathrm{mm}\right)= 100* \left(1+ \frac{76.28}{\left(611.11 * 0.00655\right)}\right)= 2005.68$$

$$\mathrm{SCD }\left(\mathrm{m}\right)= \frac{2005.68}{1000} =2.01$$

where:

*f*: 100 mm.
*A*: *RL*_p__'-__sto__'_ = 76.28.
*x*: 611.11 pixels (yellow line measurement in Fig. 8-3c).
*y*: 0.00655 mm.

The trichion (*tr*′) to menton (*m*′) distance is a favorable dimension to use for the profile view focus distance estimation, when the temporomandibular joint is at rest, since these two landmarks (1) fall close to the same plane as the palpebral fissures within the relative parallel zone of the face and (2) provide for a large inter-landmark distance that minimizes the impact of any landmark placement errors by the observer [12, 31]. However, other facial dimensions can also be used, as illustrated above. In Table 2, we demonstrate validity of the above approach using the vertical distance between stomion (*sto*′) and a chord connecting the two pupils (*p*′) for each of three authors for five separate whole integer focus distances. Note here the *p*′-*sto*′ measurement is not as large as the *tr*′-*m*′ distance, so it holds larger relative observer error, but it is useful for subjects who either have a receding hairline and/or a beard that make the trichion and menton landmarks impossible to determine (such as for the first author [CS]). We employ the *p*′-*sto*′ dimension across all three authors here to provide a conservative and robust insight on method accuracy (i.e., when shorter reference distances with higher relative observer errors must be used). We further demonstrate the validity of the methods by using a set of formerly acquired photographs from Stephan’s original study [44] to illustrate reproducibility of focus distance estimation with images taken of the same subject on different days by different photographers (in this case with four years intervening between photography sessions). A full-frame DSLR camera with a prime lens was used for all photographs.

## Focus distance estimation accuracies for profile images

### Tests using author-derived images (this paper)

The focus distance estimate from the profile view images (2–6 m) taken of three authors here and using the *PerspectiveX*/*p*′-*sto*′ combination was, on average, 0.34 m (Table 2). This represents the equivalent of 8% grand mean error. This is close to and only slightly larger than the 6% error obtained from *PerspectiveX*’s use with palpebral fissures alone for frontal images as obtained in Stephan’s [44] original validation work and the 6% error reported by Stephan and Armstrong [31] using the same 100-mm lens in the second validation study (*n* ≥ 30 subjects). The slightly increased SCD error when moving to ¾ or profile views (Table 2) derives from the use of an estimate of the real-life size of a second character in a separate photograph that is set by a mean for the palpebral fissure length, in contrast to using a directly measured sample mean for the second anatomical character. The minimum error observed for the *PerspectiveX*/*p*′-*sto*′ combination was 0.01 m for subject 3 at 2 m, and the maximum error was 1.05 m for subject 1 at 6 m.

### ***Tests using an existing image set from Stephan ***[44]

Focus distance estimation using the first author’s frontal and 30-degree rotated profile image acquired by a different photographer (JC) in Stephan’s original study [44] and the *p*′-*sto*′ measurement yielded a mean error of 0.2 m across the 10-m range that was the equivalent of 4% of the actual SCD. This is < 25% of the mean error value for the same images using just the palpebral fissure length only in rotated head views (0.8-m error) [44]. Of course, the degree of error followed the typical and expected pattern that it was larger at larger SCD in accordance with the decreased resolution of the face at these larger distances [31, 44] (see Table 3). The smallest focus distance estimation error was 0.03 m at 1 m, and the largest was 0.97 m at 10 m using the *PerspectiveX*/*p*′-*sto*′ combination (Table 3). In terms of focus distance estimation differences between the *PerspectiveX*/*p*′-*sto*′ combination and the frontal view *PerspectiveX/en*′-*ex*′ combination, the mean percent error was close to equivalent (4% and 6%, respectively).

## Discussion

Perspective has long been recognized as an important factor for characterizing 3D objects on 2D planes, not just in photography, but also in architectural illustrations where it provides depth and realism. This has been the case ever since the principles of perspective were first discovered in the Renaissance [54], and its relevance for, and accountability in, craniofacial superimposition is long overdue.

Perspective is set by the vantage point of the viewer (*x*, *y*, and *z* positions) which translates directly to camera position in craniofacial superimposition. The vantage point holds consequences for how the anatomical form of the face and skull is manifested on the 2D image plane making it a critical variable [46]. The perspective embedded in the facial photograph must be accounted for in the skull photograph if anatomical comparisons between the two images, via superimposition, are to be valid. While often attempted, it is not appropriate to ignore the perspective distortion or to re-zoom images using adjustable zoom lenses in fixed aspect ratio fashion (see, e.g., [7, 12, 40, 47, 48, 51, 52]) to try to combat the perspective distortion. When the same focal length and subject-to-camera distance is used, the images will already be at the same natural size and perspective [31, 43, 44], eliminating any requirement for image scaling. Only in the special and very specific case that the same subject-to-camera distance has been used, but a different focal length lens has been employed, is fixed aspect ratio image scaling appropriate in the craniofacial superimposition context [44].

Multiple studies have now demonstrated that the focus distance, measured from the image receptor to the subject, can be adequately estimated for frontal view facial photographs using the palpebral fissure length and the *PerspectiveX* algorithm for images taken with prime lenses on full-frame DSLR cameras [31, 44]. This study shows that when multiple images of the same person exist, including both frontal and non-frontal view images, the ratio of the measured palpebral fissure length to the sample mean can additionally be used to calibrate a secondary anatomical feature (visible in both the frontal and profile view images) to enable *PerspectiveX* to be employed for the profile images. This enables the secondary anatomical feature to be used as the input variable in the *PerspectiveX* equation, permitting the focus distance to be estimated for profile (or partial profile) images with very good accuracy that is comparable to the original *PerspectiveX/en*′-*ex*′ combination. This resolves the previous problem that the focus distance estimation was not possible for images rotated by more than 20 degrees at < 2 m focus distance or 30 degrees at > 3 m focus distance [44]. When estimating these distances using *PerspectiveX*, there is no requirement for the multiple view images to come from the same camera, lens, and/or focus distance. Rather, the focus distance estimation methods can be applied to images taken with entirely different settings, so long as the nominal focal length used for each image is known. For adult subjects, where annual growth and/or aging changes are minimal, frontal and profile images can be acquired with substantial intervening time between images (months to years). For subadults, however, this does not hold true since growth changes can be substantial, even on a yearly basis. Subsequently, frontal and profile images must be taken close together (the closer the better) and not with more than 1 year intervening between acquisition sessions.

It is imperative to emphasize that *PerspectiveX* does not provide an exact distance result that is free from error. Rather, the result is an estimate; it is likely to contain some error. In simple terms, this capacity for an approximation is better than no capability at all to estimate the focus distance. Largely, the *PerspectiveX* approximation works because there is a tolerance for error due to the reverse exponential nature of the perspective distortion curve, such that at longer focus distances, larger distance estimation errors can be tolerated [43]. In many ways, this is convenient for superimposition since longer focus distances are also associated with poorer resolutions of the face at a set focal length (i.e., the head size decreases in size in the field-of-view at longer focus distances) [31, 43]. On the other hand, much smaller raw errors at short subject-to-camera distances hold additional potential to be problematic for superimposition (even though resolutions of the face are typically better) as little tolerance exists for even small errors at such short subject-to-camera distances due to the reverse logarithmic nature of the perspective distortion curve [43]. This somewhat counterintuitive relationship is important to fully grasp, since it potentially means images acquired at a mid-focus distance range may be more ideal for superimposition comparisons.

*PerspectiveX*’s capability to estimate the focus distance on scientifically quantifiable grounds now provides the very first opportunity to derive and validate standardized anatomical criteria for evaluating the degree of correspondence between photographs of a face and a skull. This is a critical undertaking to enable the second major component of the superimposition approach—evaluation of the anatomical correspondence between skulls and faces. Previously formulated criteria using perspective non-matched images (see, e.g., [49]) must clearly be approached with a good deal of caution since resulting anatomical guidelines may not be robust. Craniofacial relationships derived using medical imaging modalities (X-ray/CT/MRI) which enable dual visualization of the skull and face in the same image offer advantages; however, these guidelines should additionally be verified in the photographic context since none of X-ray, CT, or MRI methods are photographic based. Until these investigations are undertaken, anatomical guidelines for facial comparison should be considered incomplete. The formulation of well-described, hard to confuse, and easy to implement anatomical criteria to rule in or out a series of skulls as matches based on face morphology represents an important and open domain for future research.

For successful forensic casework use of *PerspectiveX*, a coordinated approach with investigating authorities is required to obtain antemortem reference photographs suitable for the superimposition. Some photographs (e.g., those obtained using prime lenses) are clearly more suitable than others for focus distance estimation with *PerspectiveX*, and effort should be awarded to sourcing preferred images. Prior to the use of any images with *PerspectiveX*, it is pertinent to check if these photographs continue to carry manufacturer-stated raw image sizes. This avoids use of images that have been cropped and/or resampled in post-production. Cropped images should be avoided because they may give the misimpression of a face being centered in the field-of-view when in fact it was not originally. Resampled images should be avoided because their pixel sizes are altered, compared to the manufacturer’s specifications, and so will produce misleading focus distance estimates. The status of both cropping and resampling for an image can be checked by reviewing the image dimensions and pixel sizes against those of the manufacturer specifications for the camera sensor. Any mismatch in data indicates post-production manipulations and precludes the image from being used. If this is encountered during cases, other non-adjusted reference images will need to be sought by the investigating authorities.

It is important to additionally emphasize that prior to any implementation of *PerspectiveX* in craniofacial superimposition casework, the method should be validated in advance for any particular camera system/antemortem facial photograph. This is easy to achieve by using an equivalent/same camera and lens to the casework image and a test subject positioned at predetermined known focus distances to ensure the *PerspectiveX* formula functions as anticipated for the specific equipment. This test is simple and easy to conduct and only requires the same make and model of camera (not serial number) reported in the Exif metadata for the electronic antemortem image. Of course, if pre-existing validation test data exist for the relevant camera system, this step is not required. Since it is not possible to anticipate in advance what camera systems will be relevant, this step may commonly be required as the first step in most casework circumstances, at least initially. Here, it is important to note that if lenses perform with systematic error when validated, correction factors can legitimately also be applied to boost accuracy of focus distance estimates.

So far, tests using prime lenses on DSLR cameras have shown replicable results even when taken by different photographers for the same subject(s) [44] or different subjects [31, 44]. This is favorable; however, *PerspectiveX*’s replicability for practitioners in independent laboratories outside the founding unit should be established. For new users of *PerspectiveX*, special care should be taken to ensure correct placement of palpebral fissure landmarks according to the original method descriptions. This is especially important because the palpebral length measurement becomes a multiplier with pixel size in the *PerspectiveX* equation, meaning that any error in palpebral fissure measurement is magnified within the calculation for the focus distance. The exocanthion (*ex*′) must be placed on the very external lateral edge of the eyelid’s rim (on the same side as the lower lid lashes) and at the geometrical vertex of the upper and lower lids, not on the adjacent side of the lower lid rim adjacent to the eye sclera [31, 44]. With regard to the medial canthus, the endocanthion (*en*′) must be placed at the medial most apex of the open fissure along the sharply defined rim, not on the caruncle or near the sclera of the eye and not on the end of any shallow groove between the medial canthal ligaments that may project beyond the open eye fissure slit [31, 44]. Since the *en*′ is not marked by a clearly defined geometric vertex like the *ex*′, but rather falls on a notch where the upper and lower lids meet medially, the apex for the *en*′ must be identified at the mid-point of the notch, where it is deepest along the eye fissure’s longitudinal axis. Special attention must be paid to these definitions since they are much more precise than usual generic definitions of the *en*′/*ex*′, namely: the corners or commissure of the eye fissure [55, 56]. Prior to casework, we recommend practitioners familiarize with landmark placement using images of known subjects in high and low image resolution conditions and with extensively pre-validated camera systems (such as Canon 6D combination with 100-mm macro prime lens [31, 44]), so that analysts can cross-check and reverify their own focus distance results following landmark placement against known ground truths. In our laboratory’s experience, newcomers to the technique often tend to place palpebral fissure landmarks too close together when confronted with the eye’s non-angular gently curving surfaces at high magnification, in part as a result of being influenced too strongly by traditional nonspecific definitions of *en*′ and *ex*′.

The entire absence of any other focus distance estimation method in the superimposition domain suggests that *PerspectiveX* should be employed as a standard, so that the focus distance does not need to be subjectively guessed. Further validation tests of the focus distance estimation method will be useful; however, the question is not one of whether focus distance estimation is possible—the mathematical principles of the method are well demonstrated [31, 44]—but rather how replicable methods are across samples, cameras, investigators, and laboratories. These studies must be pursued in the future.

Like most technical methods, *PerspectiveX* is not without its limitations, and there will be circumstances where the method cannot be employed due to circumstances of the case. For example, there are some photographs that lend themselves well to focus distance estimation using *PerspectiveX* (mid-range high-resolution images taken with prime lenses) and others that do not (short-range images taken with zoom lenses set to the longest focal lengths). In other words, *PerspectiveX* is not a silver bullet to resolve the focus distance employed for any and all photographs that might be available for superimposition; instead, it should be used with strategically selected images. For forensic cases where identities of skeletons are sought by superimposition methods, it is integral for investigating authorities to seek and obtain the best antemortem facial photographs to use for the superimposition procedure, including the *PerspectiveX* focus distance estimation. Presently, these criteria include the following:

• Raw-state electronic (not wet-film) images free from cropping or pixel size resampling.
• Images taken using DSLR camera body with a prime lens or other readily available camera/lens combination that can be tested for *PerspectiveX* suitability prior to casework employment.
• Images with high resolution and good focus.
• Multiple images of the subject with the face exhibited from different views (e.g., frontal, profile, and three-quarter).
• Images with the face centered (or near centered) within the field-of-view/image sensor.
• The plane of the face should be orthogonal to the line of sight of the camera for frontal views (use the visibility of the ears to help judge whether the face is orientated directly at the camera).
• Images of the face with anterior teeth displayed in open-mouth pose (e.g., large smile).
• Images that are not taken at the extreme focus distance ranges for the lens at hand (i.e., very close or very far away, but rather are mid-range).
• Recent images taken soon before death. This is especially relevant to superimpositions concerning subadults (females ≤ 16 years of age and males < 23 years of age) since their growth is not complete and changes rapidly with time [53, 57].
• Images of the face with the palpebral fissure clearly visible and unobstructed by any headwear or spectacles.

For images that meet the ideal conditions mentioned above, there are limitations inherently embedded in the *PerspectiveX* approach that cannot be avoided, and these must be explicitly acknowledged. That is, *PerspectiveX*:

1. Applies the simple thin lens model of photography to all images, even for far more complex camera systems that use multiple lens elements
2. Regards the distance between the subject to the front nodal point (*d*–*f*) as a generalization of the working distance (*d*) in front of the lens
3. Regards the focus distance (*f*′) to be a generalization of the image distance (*d*′)
4. Uses the manufacturer’s stipulated (and often rounded) nominal focal length for the effective focal length value of the lens
5. Uses 2D in-photo measurements of palpebral fissure lengths from frontal images as equivalents of 3D palpebral fissure length measurements, even though the two are technically not the same (the palpebral fissure wraps around the eyeball laterally such that its *ex*′ is placed more posteriorly than the *en*′, providing a longer measurement in 3D than in 2D)
6. Ideally requires orthogonal alignment of the face to the camera for frontal images (this may not be attained if the faces possess slight rotations in the frontal view even if the subject’s eyes are directed towards the camera)
7. Uses anatomical landmarks for facial feature measurement that may be associated with observer errors for placement
8. Uses the palpebral fissure length mean as a proxy for individual’s real-world values, because the latter are unknown

Irrespective of these small errors which compound when using *PerspectiveX*, validation tests show the grand errors are not enough to invalidate the overarching focus distance estimation result. In the craniofacial superimposition context, this is facilitated by the reverse logarithmic nature of the perspective distortion curve and a tolerance window that enlarges for longer focus distances [43].

**Fig. 9:**
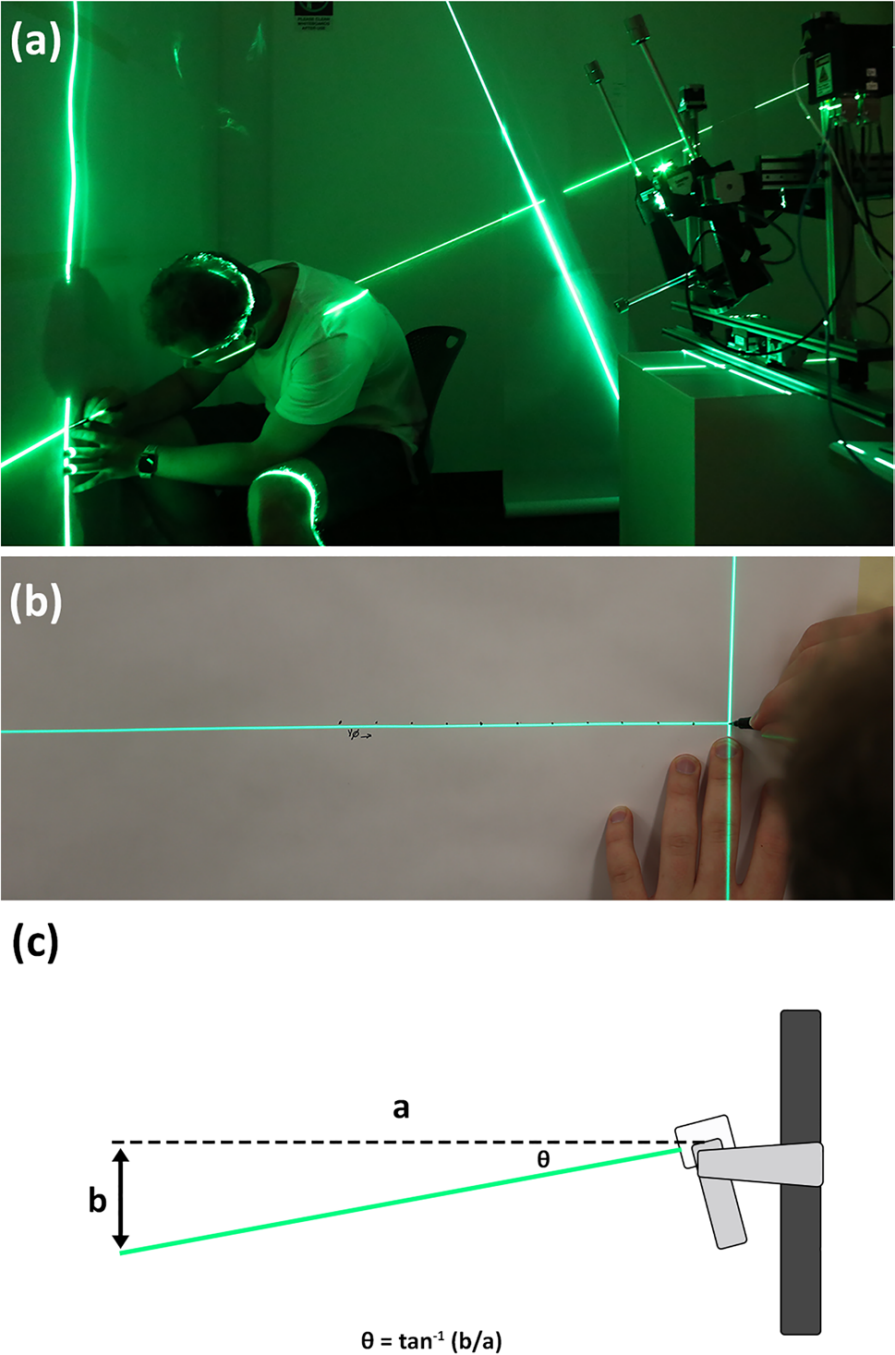
Validation testing of coordinate positioning accuracy of a superimposition skull device using a 3-way cross laser. **a** Three-way cross laser in the skull position device, with laser projection to two vertical walls, to measure angular accuracy of the device. **b** Marking the center position of the laser-cross for trigonometric calculation of device step angles and ranges (here, *x*- rather than *z*-axis travel is mapped). **c** Illustration of the angle derivation using the inverse tan function at distance of 1273 mm (a) yielding 10 mm spacing of the laser-cross (b) and providing quantification of the angular precision (θ) to 0.45 ± 0.05 degrees

It must be further noted that while the methods described in this paper provide the first ability to resolve perspective issues in craniofacial superimposition for both frontal and non-frontal (profile) views, this action alone does not resolve all of the major lingering gaps within present-day craniofacial superimposition methods to produce what might be considered an all-inclusive fully fledged scientific protocol for craniofacial superimposition. As already mentioned, there is a necessity to derive valid (perspective matched) anatomical assessment criteria, which are specifically applicable to photographs to enable analysis of the degree of match between skulls and faces. In addition, the validity of the superimposition result hinges on capabilities of skull positioning devices to accurately orientate skulls for video and/or still-frame photography. While many mechanical skull positioning devices have been previously presented in the literature [7, 8, 30, 58–61], their angular step resolution and associated material property and operational errors have not been adequately documented. Here, it is important to note that manufacturer-produced technical specifications of motors to drive mechatronic rigs are not sufficient accuracy data alone for validation as the rig design, rig materials, and rig construction may all impair overall instrument performance. Validation tests of whole-of-device systems are required in advance of being used in superimposition protocols. Tests of the accuracy of skull positioning rigs are not especially difficult to undertake; rather, they simply have not yet been paid adequate attention. Degrees of angular placement precision can, for example, be easily measured by inexpensive trigonometry-based methods where a 3-way cross laser is placed in the skull clamp (Fig. 9). Here, the distance of the skull positioning device to the measurement surface sets the resolution with which angular movement can be measured, so it can readily be adjusted to obtain the desired degree of sensitivity for error measurement.

While craniofacial superimposition has previously been described as a straightforward, long-tested, and well-accepted procedure that is accurate [3, 7–9, 40, 41], the method is in reality a much more finicky and technical undertaking that at present lacks key validation components. While several technological advances have provided substantial prior method improvements, see e.g., [3, 9, 11, 32, 62–67], technology alone has not resolved the full suite of superimposition’s underlying weaknesses. Perspective distortion is one such integral issue that (1) has long been recognized in the superimposition domain [12, 42, 46–48], (2) has been deemed too difficult to resolve [12, 47, 48], and (3) applies to all superimposition approaches whether they be traditional video approaches using real skulls [3, 11, 62–64] or newer fully computerized approaches that use surface meshes of skulls [32, 65–67]. This paper provides the first comprehensive solution to the perspective distortion conundrum by extending the *PerspectiveX* algorithm to both frontal and profile views. This enables a scientifically grounded focus distance estimate to be used for skull photography, ensuring subsequent morphological comparisons between the skull and the face are anatomically valid.

**Table 1:** Basic classifications of prime lenses by their focal length/angle of view

Lens category	Focal length (mm)	Typical angle of view for the focal length (degrees)
Ultrawide angle	< 24	84–114
Wide angle	24–35	63–84
Standard angle	36–50	47–62
Telephoto	50–199	12–46
Super telephoto	> 200	< 12

**Table 2:**
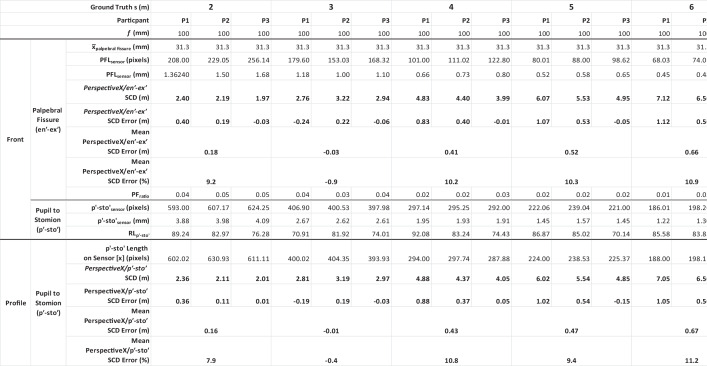
SCD estimation for profile view facial images (see, e.g., Fig. 8 panels 1–3) taken at whole integer values between the ground truth distances of 2–6 m using a Canon 6D full-frame 20.2-megapixel camera body fitted with a Canon IS 2.8-speed 100-mm fixed macro lens. Photographer and image measurer was the second author (SH)

**Table 3:**
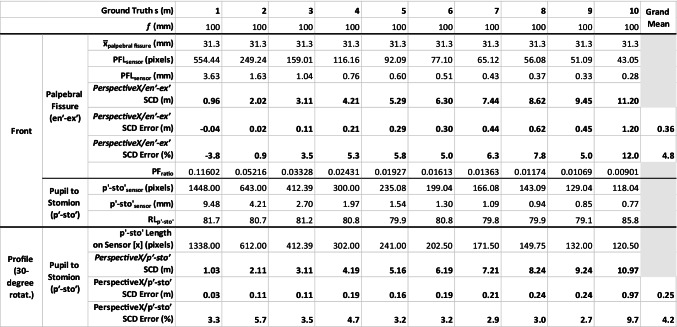
SCD estimation for profile view facial images (see, e.g., Fig. 8 panel 4) taken at whole integer values between the ground truth distances of 1–10 m using a Canon 6D full-frame 20.2-megapixel camera body fitted with a Canon IS 2.8-speed 100-mm fixed macro lens. Photographer was Jodi Caple [44] and image measurer was the first author (CS)
